# Studying mutation rate evolution in primates—the effects of computational pipelines and parameter choices

**DOI:** 10.1093/gigascience/giab069

**Published:** 2021-10-21

**Authors:** Susanne P Pfeifer

**Affiliations:** School of Life Sciences, Arizona State University, Tempe, AZ 85281, USA; Center for Evolution and Medicine, Arizona State University, Tempe, AZ 85281, USA; Center for Mechanisms of Evolution, Arizona State University, Tempe, AZ 85281, USA

**Keywords:** primates, de novo mutation, germline mutation rate, false-positive rate, false-negative rate, computational pipeline

## Abstract

This commentary investigates the important role of computational pipeline and parameter choices in performing mutation rate estimation, using the recent article published in this journal by Bergeron et al. entitled “The germline mutational process in rhesus macaque and its implications for phylogenetic dating” as an illustrative example.

## Background

The handful of non-human primate germline mutation rate studies published to date [1–7] are largely similar with regards to their experimental design—using PCR-free library preparation protocols (except [2]), followed by 100–150 bp paired-end Illumina sequencing. They also share similar bioinformatic pipelines—aligning reads to a species-specific reference assembly, marking (or removing) duplicates, performing a base-quality score recalibration to improve variant detection (except [1, 4, 6]), and calling variants using the Genome Analysis Toolkit (GATK) [8, 9] (except [1]). This procedure is then followed by an identification of *de novo* mutation candidates via the detection of Mendelian violations (e.g., sites at which the offspring is heterozygous despite both parents being homozygous for the same allele). However, several important differences exist in the estimation of the number of loci at which genuine *de novo* mutations can be identified (often referred to as “callable sites”, which are part of the denominator in the rate estimation), as well as the computational filters developed to mitigate false-positive and false-negative results. Importantly, these differences have led to datasets that are exceedingly difficult to compare.

Recently, Bergeron et al. [10] used genome-wide sequencing data from 19 rhesus macaque (*Macaca mulatta*) trios to estimate a mean spontaneous germline mutation rate of 0.77 × 10^–8^ per base pair per generation, which is 32.8% higher than the per-generation rate of 0.58 × 10^–8^ per base pair previously reported by Wang and colleagues for the species [6]. This difference is likely driven by a combination of factors, including (i) biological (e.g., parental ages at puberty and reproduction), (ii) experimental (e.g., the design of the study by Bergeron et al. differs from most of the aforementioned work by amplifying samples using PCR—a process that introduces errors ascribed to both mistakes made by the polymerase as well as thermal damage—followed by sequencing on a BGISEQ-500 platform); and (iii) methodological (e.g., user-defined criteria, as is the focus here).

## Quantifying the Impact of Variability in User-Defined Criteria

Most primates studied to date exhibit mutation rates of ∼10^–8^ per base pair per generation. Thus, identifying the ∼70 newly arising mutations in an individual genome [11] can resemble the proverbial search for needles in a haystack. Complicating this search are technical artifacts resulting from amplification biases, the inevitable presence of sequencing errors (which occur at rates that are ∼2 orders of magnitude higher than primate mutation rates), as well as the bioinformatic pipelines used to process the high-throughput sequencing data generated. Accurately distinguishing genuine *de novo* mutations from artifacts thus requires the application of stringent computational filter criteria. As such, choices of filtering criteria and their thresholds can have profound impacts on the resulting mutation rates estimated.

**Figure 1:**
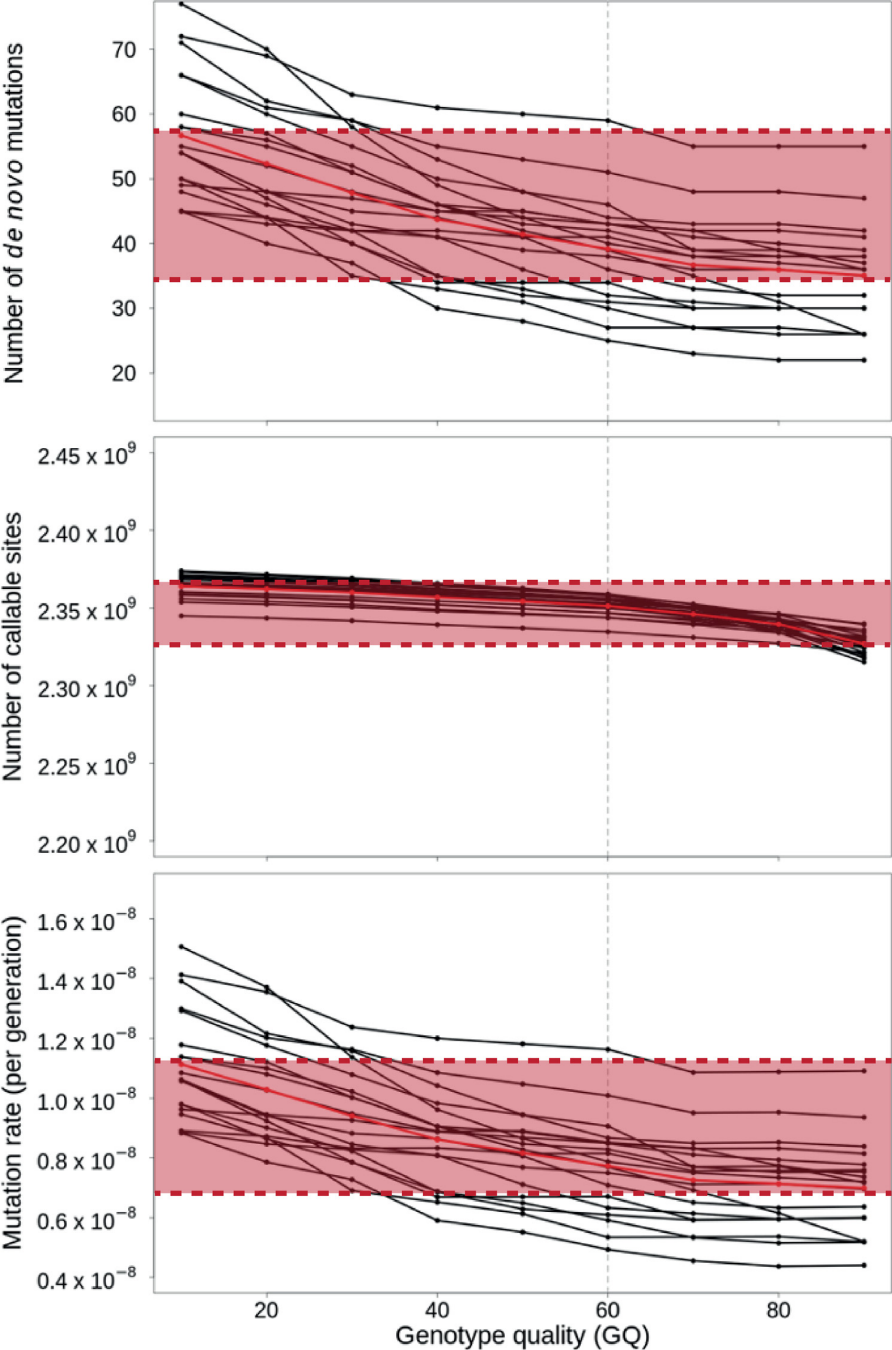
Influence of genotype quality (GQ) thresholds on mutation rate estimates. Bergeron et al. found that varying their GQ threshold from 10 to 90 led to mean mutation rate estimates ranging from >1.1 × 10^–8^ to <0.7 × 10^–8^ per base pair per generation in their trios (highlighted in red), as adapted from Bergeron et al. [10]. Because this criterion can lead to systematic biases in rate estimates, rather than choosing this filtering threshold arbitrarily, the sensitivity and specificity of the detection pipeline should be evaluated using simulations and/or benchmarking datasets for which the “ground truth” is known.

To highlight just 1 example, Bergeron et al. found that varying their genotype quality (GQ) threshold from 10 to 90 identified between ∼55 and ∼35 *de novo* mutations per trio, leading to mean mutation rate estimates ranging from >1.1 × 10^–8^ to <0.7 × 10^–8^ per base pair per generation in their trios (Fig. 1). The well-established and commonly used GATK Best Practices pipeline for variant filtering [8]—the software used by the authors to call, genotype, and filter variants—recommends a GQ threshold of ≥20 to obtain high-quality genotype calls [12]. The same genotype quality cut-off of GQ ≥20 has also been recommended in the recently published guidelines for the identification of *de novo* mutations in studies of rare human disease [13]. In contrast, the results presented by Bergeron and colleagues are based on a GQ threshold of ≥60—a higher threshold that may serve to increase the confidence in the assigned genotypes. While high thresholds may sound advantageous, the systematic biases in rate estimation observed by the authors demonstrate the need to justify a specific GQ filtering threshold for any given dataset. Ideally, this justification would include simulations incorporating realistic sequencing error models, for which the “ground truth” is known, thus allowing for the investigation of the effects of different GQ thresholds on the sensitivity and specificity of the detection pipeline. Such a justification is lacking in their study, rendering their choice largely arbitrary. Benchmarking studies against large, well-characterized datasets can also provide important insights, however the validation performed by Bergeron et al. using a single chimpanzee trio (previously published by several of the authors) is less useful in this regard, because (i) the previous study used a similarly high GQ threshold (≥65) [5] as their own pipeline (≥60) and (ii) previously identified *de novo* mutation candidates were not independently validated by an orthogonal technology (e.g., by Sanger sequencing all genotypes of the trio).

**Figure 2:**
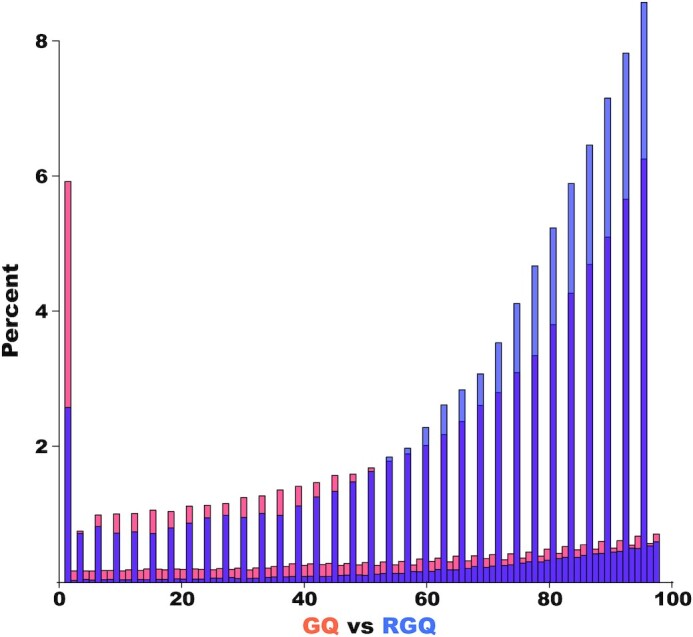
Histogram of genotype quality (GQ) and reference genotype quality (RGQ) scores of 1 million variant and invariant sites called in one of the rhesus macaque trios of Bergeron et al. [10] (excluding sites ≥99). The 2 annotations are calculated differently by GATK [8, 9]; thus it is inappropriate to consider them as equivalent when filtering variants and estimating the callable genome, respectively.

To obtain a mutation rate per base pair per generation, the number of *de novo* mutation candidates (corrected by the false-positive rate) is divided by (2x, in diploids) the number of sites at which genuine *de novo* mutations could have been identified (corrected by the false-negative rate). To determine the number of callable sites, Bergeron et al. used GATK's HaplotypeCaller in BP_RESOLUTION mode to obtain annotations for each site in the genome (variable and invariable), keeping only those sites that passed their filters. Although this strategy is expected to perform well for the majority of their selected filtering criteria, GQ thresholds are an important exception. Specifically, GATK genotype quality scores at invariant sites (referred to as “reference genotype quality” or “RGQ” in GATK 3) are calculated differently than those at variant sites and are thus not directly comparable (see Fig. 2). Consequently, different thresholds need to be applied to variant and invariant sites to avoid any bias in downstream analyses—a consideration neglected by the authors.

**Figure 3:**
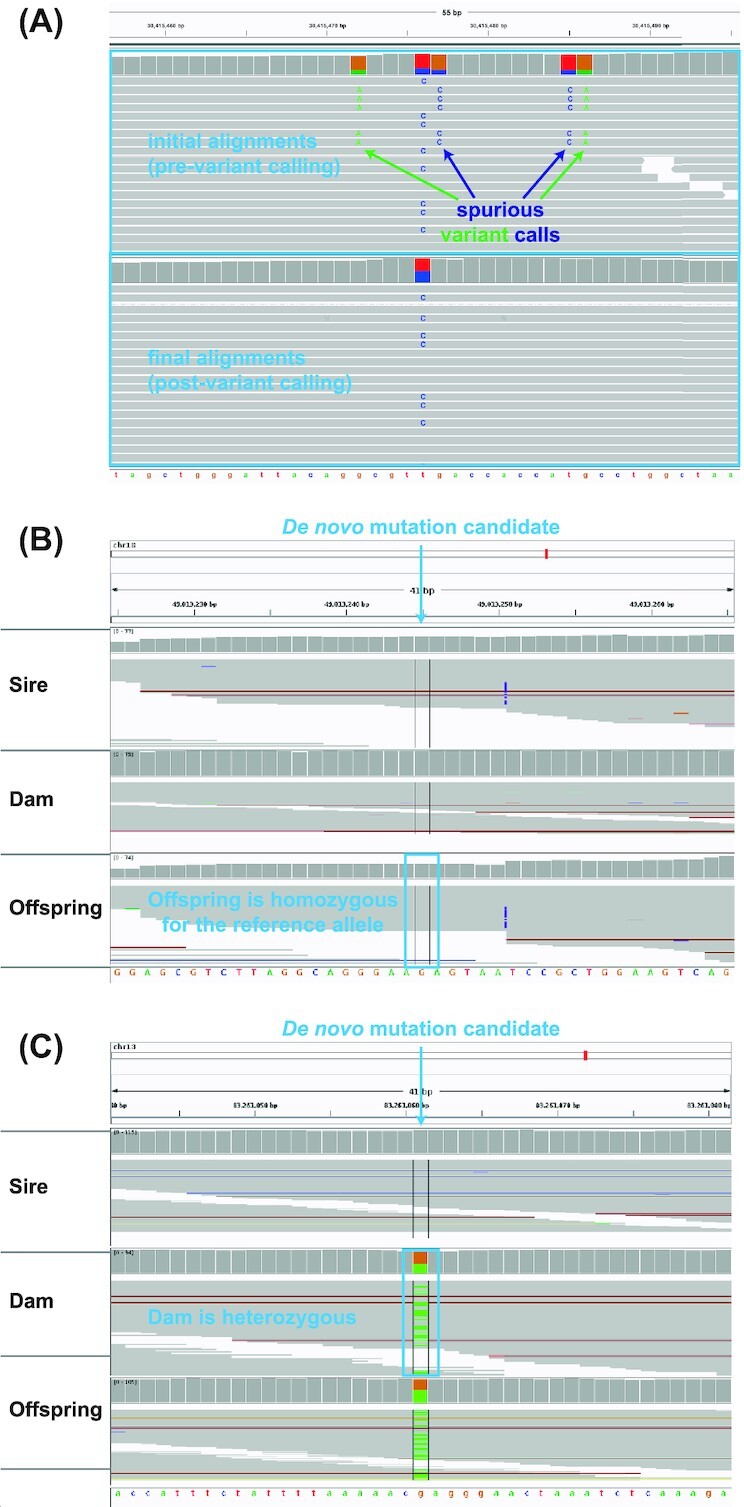
Manual (visual) curation of *de novo* mutation candidates. (A) Initial read alignments (pre–variant calling) can differ from the final (post–variant calling) alignments. (B, C) Two *de novo* mutation candidates identified by the computational pipeline of Bergeron et al. that fail the definition of a *de novo* mutation. The example in panel (B) shows no evidence for the alternative allele (mutation) in the offspring, while that in panel (C) shows that the dam is heterozygous at the locus of interest and could hence have passed on the mutation to the offspring (adapted from Bergeron et al. [10])—illustrating the pitfalls of their approach of basing the false-positive rate estimation on alignments obtained before rather than after variant calling.

More generally, Bergeron et al. [10] based their filtering thresholds on a manual (visual) curation of their *de novo* mutation candidates. During variant calling, GATK's HaplotypeCaller locally re-assembles genomic regions to determine potential haplotypes and re-aligns the reads to those that are most likely [9]. The authors visually explored their 744 candidate *de novo* mutations both before and after variant calling, excluding 81 and 50 variants as false-positive calls, respectively (corresponding to false-positive rates of 10.9% and 6.7%). Because initial read alignments can differ from the final alignments (see Fig. 3A), concordance between the 2 inspected datasets was poor (with an overlap of 47 variants [56%]). Problematically—and rather perplexingly—the authors based their analyses on the dataset obtained from the visual inspection of the initial (i.e., pre–variant calling) alignments. For the sake of illustration, Figs 3B and C show 2 sites that have been identified as *de novo* mutation candidates by the computational pipeline of Bergeron et al., despite failing the definition of a *de novo* mutation—either because the offspring is homozygous for the same allele that is carried by both parents (in other words, there is no mutation at that site; panel B) or because 1 of the parents is heterozygous at the locus of interest and could hence have passed the mutation on to their offspring (panel C). This, combined with other results found in their Supplementary Material, suggest 2 possibilities. Either there exist systematic issues in their computational pipeline that resulted in the (incorrect) inclusion of candidate sites that do not constitute Mendelian violations, or their computational pipeline correctly identified Mendelian violations but they are simply unable to discern between genuine *de novo* mutations and false-positive calls based on the initial alignments as reads were re-aligned during the variant calling process. Assuming correct pipeline implementation, Fig 3 suggests that visual inspections need to be performed on the final alignments in order to accurately estimate and account for false-positive calls. For example, quite apart from the variation due to the GQ threshold noted above, simply using the final alignments under the authors' implementation would itself result in a 5.2% higher mutation rate estimate for the species (0.81 × 10^–8^ per base pair per generation).

Finally, in addition to considering false-positive rates, it is equally important to carefully estimate the rate of false-negatives—i.e., genuine *de novo* mutations that have either not been identified or that failed one or more of the applied filtering thresholds. Amongst their callable sites (i.e., positions that survived filtering, including the strict genotype filtering threshold), Bergeron et al. [10] estimated the false-negative rate of their study as the proportion of genuine heterozygote sites (i.e., heterozygous sites in the offspring for which the parents were homozygous for 2 different alleles) that failed their site and allelic balance filters. In so doing, the authors neglected to account for several other sources of false-negative calls. Specifically, given that their method operates on the variant call set, the authors are unable to take into account any false-negatives that arose due to errors in the earlier steps in their discovery workflow, including read alignment and post-alignment processing. The matter is further complicated because the authors cannot actually evaluate a “ground truth” dataset—in other words, a site appearing as a heterozygote might in fact not be heterozygous. Given the many different steps involved in the identification of *de novo* mutations (alignment, post-alignment processing, variant calling, and filtering), it is thus essential to take into account the impact of the entire computational pipeline in order to obtain a realistic estimate of the false-negative rate. This approach has been successfully implemented in earlier studies by simulating synthetic mutations via allele-dropping according to characteristics drawn from distributions observed in the specific dataset [e.g., 1, 2, 7]. Moreover, unlike in the method applied by the authors, the positions of all synthetically generated *de novo* mutations are known *a priori* in these simulations, thus allowing for reliable performance evaluations and hence parameter justifications.

## Conclusions

The study conducted by Bergeron and colleagues [10] highlights the profound, far-reaching influences that computational pipeline and parameter choices can have on mutation rate estimates. Although a much-needed community-level consensus on “gold standard” computational pipelines for pedigree-based mutation rate estimation remains elusive (not least due to differences in study design), the choices made in individual studies still require justification and the range of possible mutation rates resulting from these choices require quantification. When left unjustified owing to a lack of ground-truthing, these choices are essentially arbitrary, resulting in a series of highly interesting datasets across primates that cannot be meaningfully utilized for comparative genomic analysis owing to their lack of equivalency.

## Editor's Note:

Several recent studies by different groups present data on mutation rate estimation for primates derived from pedigree sequencing. Within this active and new field, a range of analysis methods are being used. As the review process of Bergeron et al. [10] has shown, there are different views regarding the choice of particular methods and pipelines. Following up on the review process, this article is part of an exchange of opinions between one of the reviewers (this commentary) and the authors [14], in the spirit of contributing to the development of consensus in this rapidly developing area of research.

## Data Availability

Not applicable.

## Abbreviations

bp: base pairs; GATK: Genome Analysis Toolkit; GQ: genotype quality.

## Competing Interests

The author declares that they have no competing interests.

## Funding

This work was supported by the National Science Foundation CAREER Grant DEB-2045343 to S.P.P. The funder had no role in study design, data collection, and analysis, or preparation of the manuscript.
